# Neurology and headache medicine in Malawi: territory, education, and the path to strengthening primary care in sub-Saharan Africa

**DOI:** 10.3389/fneur.2026.1869988

**Published:** 2026-10-05

**Authors:** Daniele Martinelli, Massimo Leone, Cristina Tassorelli

**Affiliations:** 1 Headache Science and Rehabilitation Unit, IRCCS Mondino Foundation, Pavia, Italy; 2 DREAM program, Community of Sant’Egidio, Rome, Italy; 3 Global Neurology Program, International Affairs, IRCCS C. Besta Neurologic Institute, Milan, Italy; 4 Department of Brain and Behavioural Sciences, University of Pavia, Pavia, Italy

**Keywords:** education, headache, IGAP, neurology, sub-Saharan Africa, task sharing, task shifting, Malawi

## Abstract

Sub-Saharan Africa (SSA) bears a disproportionate burden of neurological and pain-related diseases while facing a severe shortage of trained specialists. In this context, expanding neurology and, particularly, headache medicine, through education and integration within primary care represents both a necessity and an opportunity. This Perspective proposes a context-sensitive framework in which three interconnected domains—territory, culture, and capacity—shape the accessibility, acceptability, and sustainability of neurological care. Drawing on the experience of the International Headache Society (IHS)–DREAM healthcare network partnership in Malawi, we examine how locally adapted education, task-shifting and task-sharing can contribute to capacity building while highlighting the limitations of approaches that focus on training alone. We argue that closing the neurological treatment gap requires a shift from specialist-centred to system-centred neurology, integrating workforce development with primary care, culturally grounded community engagement, appropriate supervision and referral pathways, supportive national health policies, and equitable access to essential medicines. Task shifting and task sharing between physicians and non-physician clinicians may constitute a sustainable component of this model when appropriately regulated, supervised, remunerated, and integrated with specialist support and referral pathways. Rather than proposing a model for direct transfer across SSA, this Perspective advocates locally led, context-dependent implementation strategies capable of adapting to diverse health systems. Such an approach reframes global neurological capacity building from the episodic transfer of expertise towards the development of resilient and sustainable systems of care.

## Introduction

The neurological revolution of the past decade has reshaped global health priorities. The *Global Burden of Disease (GBD) 2023* study reports that neurological disorders account for 443 million disability-adjusted life years (DALYs), representing 16.8% of the global disease burden—a figure that has doubled since 1990. Stroke, migraine, epilepsy, dementia, neuropathies, and chronic pain dominate this spectrum (1). Sub-Saharan Africa (SSA) is markedly affected: it contributes nearly one-quarter of the global neurological DALYs. A challenging problem, considering that across the different SSA territories fewer than 1,000 neurologists practise, corresponding to 0.043 neurologists per 100,000 inhabitants, compared to more than 7 per 100,000 in high-income regions (2). Headache disorders deserve explicit attention within neurological care because they are among the most prevalent and disabling neurological conditions worldwide (1, 3). In 2023, an estimated 2.9 billion people were affected by headache disorders globally, with migraine accounting for most headache-related disability despite tension-type headache being more prevalent (4). Headache burden falls disproportionately on women and on people in their most productive years; in GBD estimates up to 2021, migraine and tension-type headache together accounted for 11.2% of all years lived with disability among women aged 15–49 years, and more recent analyses continue to show markedly higher YLD and DALY rates in females (5).

In many low- and middle-income settings, the main problem is not only the frequency of disease but also the limited access to effective headache care (6). Headache care pathways are often poorly organised, diagnosis is delayed or missed, and many patients never reach appropriately trained providers despite the availability of evidence-based acute and preventive treatments, ranging from widely accessible analgesic and non-steroidal anti-inflammatory drugs to preventive options, such as beta blockers, tricyclic antidepressant and antiepileptic drugs (7).

In 2022, the World Health Organisation (WHO) launched the *Intersectoral Global Action Plan on Epilepsy and Other Neurological Disorders (IGAP, 2022–2031)* (8). This framework establishes five strategic objectives, with education serving as a transversal pillar underpinning all of them. Education enhances public awareness and stigma reduction, expands training opportunities for healthcare professionals, strengthens policy prioritisation, and promotes inclusivity in health through interdisciplinary collaboration (9). These aims echo the *UNESCO Global Education Monitoring Report 2020*, which emphasises inclusive and equitable education as a social determinant of health (10).

Neurology education in this context is not simply a technical requirement, it is a determinant of health equity. The evidence linking education to life expectancy, morbidity, and socioeconomic status is well established. (11) In many low-resource settings across SSA, where more than 60% of the population lives in rural areas (12), education is key to connecting geography, healthcare structure, and access to neurological care. The challenge lies in transforming education into actionable capacity at the level of primary care (PC), where most health encounters occur (13).

## Territories and health systems

The impact and reach of medicine depend heavily on territory. The 1.4 billion inhabitants of SSA are distributed across a highly diverse group of countries and territories, encompassing densely populated coastal cities alongside remote rural regions. The *Atlas of African Health Statistics 2022* shows that in 2019, among the six WHO regions, Africa was fifth in terms of the share of the gross domestic product (GDP) spent on health, with 5.3%. The European Region led with 7.6%, while South-East Asia came last with 4.4%. The African continent’s population represents 14.4% of the world’s people. Still, it accounts for only 1% of the global health spending, with investments, facilities and expertise mostly concentrated in the cities/metropolitan areas. Access to neurology and pain medicine is consequently governed by geography. Regional averages obscure marked inter-country variability in healthcare financing, disease burden, specialist workforce density, educational capacity, and the organisation of health services. Consequently, the opportunities and barriers to improving neurological care differ substantially across settings, and interventions that prove effective in one country cannot be assumed to be directly transferable to others without careful adaptation to local epidemiological, socioeconomic, and health system contexts. As of 2020, Kenya could count on about 25 neurologists for 55 million inhabitants, Nigeria has around 100 for more than 242 million, and Malawi has only two full-time neurologists for nearly 22 million citizens (14, 15). Even greater disparities are evident in pain-medicine services, not only in terms of access to care or healthcare professionals, but also in terms of limited access to drugs (16). For millions of Africans, neurology remains inaccessible both geographically and financially, demanding an urgent call to action (17). Analysing the healthcare situation in SSA from the standpoint of the pyramidal model further amplifies the impact of territorial issues (Figure 1).

**Figure 1:**
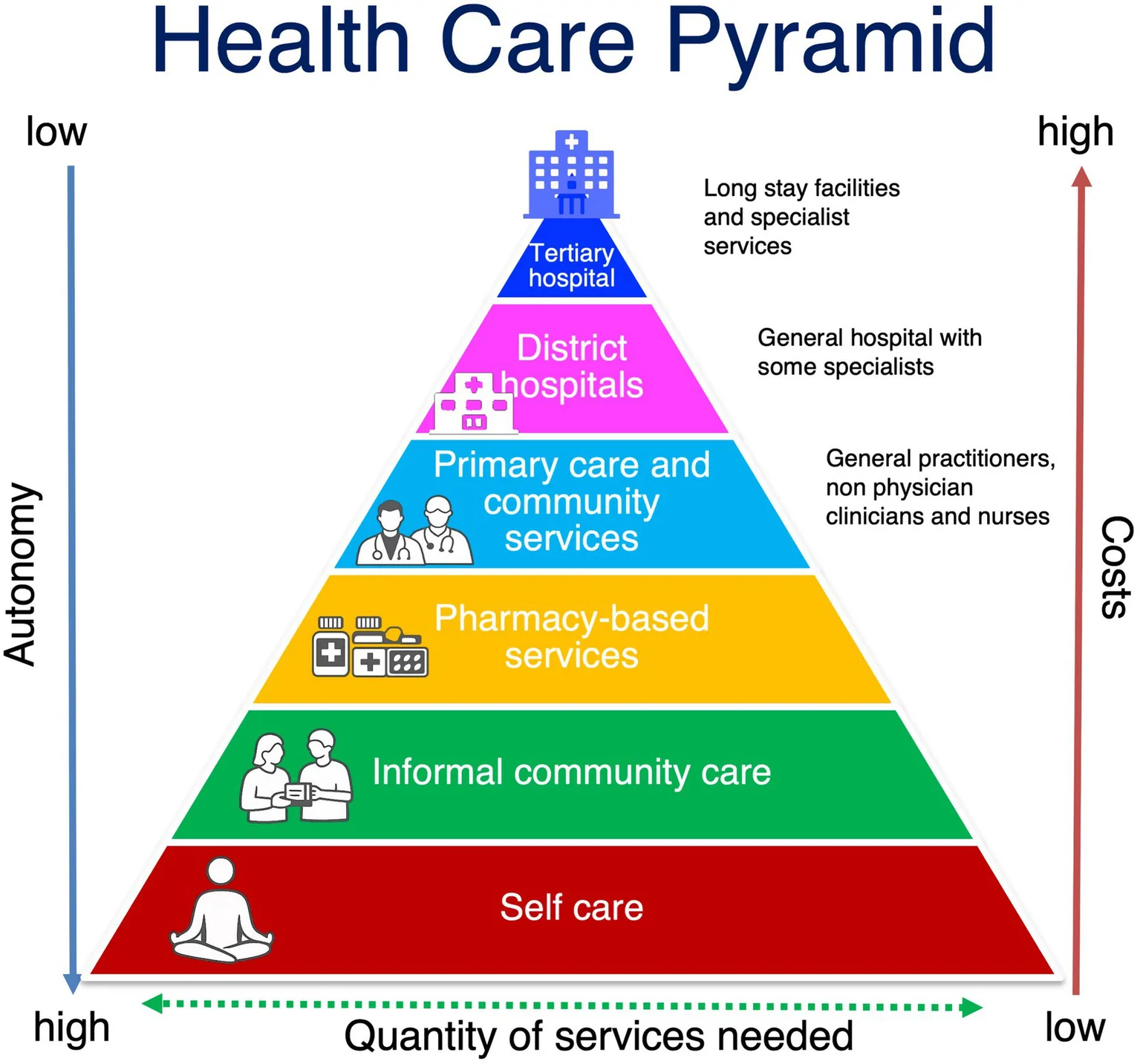
Health care system pyramid. It represents the levels of medical care or public health interventions based on their frequency, complexity, cost, and population impact. It is structured with the most common, foundational services at the base and specialised, rare tertiary services at the top.

In SSA, community health workers, pharmacists and nurses form the base, serving as the first point of contact for preventive and basic care services. Above them are non-physician clinicians (NPCs), distinct health care providers peculiar to the African continent, introduced originally as a response to communicable diseases (CDs). They undergo three to 4 years of training, which qualifies them to perform many of the diagnostic and therapeutic functions of physicians (18). At the top stand medical officers and specialists, concentrated in tertiary centres. The efficiency of this pyramid depends on its base, yet neurology has historically been confined to its apex. Moreover, examining the healthcare pyramid reveals a paradox: the greatest expertise is found at the top, while the greatest number of patients is found at the base. True efficiency requires a shift of focus, with intellectual and financial investment being redirected towards the lower tiers.

PC is therefore both the foundation and the bottleneck of neurological access. It is here that headache, seizures, neuropathic pain, and post-stroke disability are first seen, but often unrecognised as neurological conditions. Many patients attribute symptoms to spiritual or traditional causes before seeking hospital care. Across SSA contexts, the distinction between psychiatric, neurological, and supernatural illness might be blurred in local explanatory models, with symptoms commonly attributed to witchcraft, spirits, curses, or ancestral forces (19–21). These beliefs are associated with fear, social exclusion, and stigmatisation of affected people, including those with mental illness, dementia, and epilepsy (20, 22). Local traditional and faith healers remain trusted health actors because they are culturally meaningful, accessible, and may be perceived by patients and communities as providing explanations of illness that are more closely aligned with local beliefs and understanding that those offered within formal healthcare settings (23). As a result, traditional healing practices commonly coexists with formal healthcare and strongly shapes patient interpretations of symptoms, help-seeking behaviors and pathways to treatment (19, 21). To strengthen neurological access, it is essential to engage this cultural dimension, transforming community leaders into allies for awareness and stigma reduction.

Cultural change, therefore, is as important as infrastructural reform. Strengthening PC requires not only improving clinical capacity, but also building trust and recognising the culturally meaningful explanatory models that shape health-seeking behaviour. Communities should be supported in viewing primary healthcare centres as accessible and trusted sources of neurological and brain-health care, while traditional and faith-based healers could be engaged as potential partners in community education, recognition of neurological symptoms, stigma reduction, and appropriate referral. Such reciprocal engagement might help connect culturally rooted understandings of illness with safe and effective biomedical pathways, without imposing the displacement of existing beliefs or practices. Education plays therefore a double role: enhancing the competence of providers and elevating the credibility of biomedical neurology in the public eye.

In this variegated context, IGAP recognises that neurological care cannot be implemented through a standardised strategy. By focusing on territories, IGAP calls for an approach that adapts to the diversity of geographical, infrastructural, and cultural contexts across regions. It explicitly identifies the territorial level, namely PC, as the foundation for achieving equity. This territorial implementation aims to address disparities in access between urban and rural areas, tailor solutions to local resources and capacities, and ensure equitable distribution of neurological services within countries. By empowering PC networks and integrating neurology into community-based health systems, IGAP intends to promote a shift from centralised, hospital-dependent models to decentralised, people-centred frameworks capable of delivering high-quality care, regardless of location.

## Task-shifting and task-sharing in neurology

The shortage of neurologists in SSA made it necessary to redistribute healthcare responsibilities in an innovative way. Two complementary strategies—task-shifting and task-sharing (as defined in Figure 2)—have been progressively implemented to manage the gap between demand and specialist availability (24).

**Figure 2:**
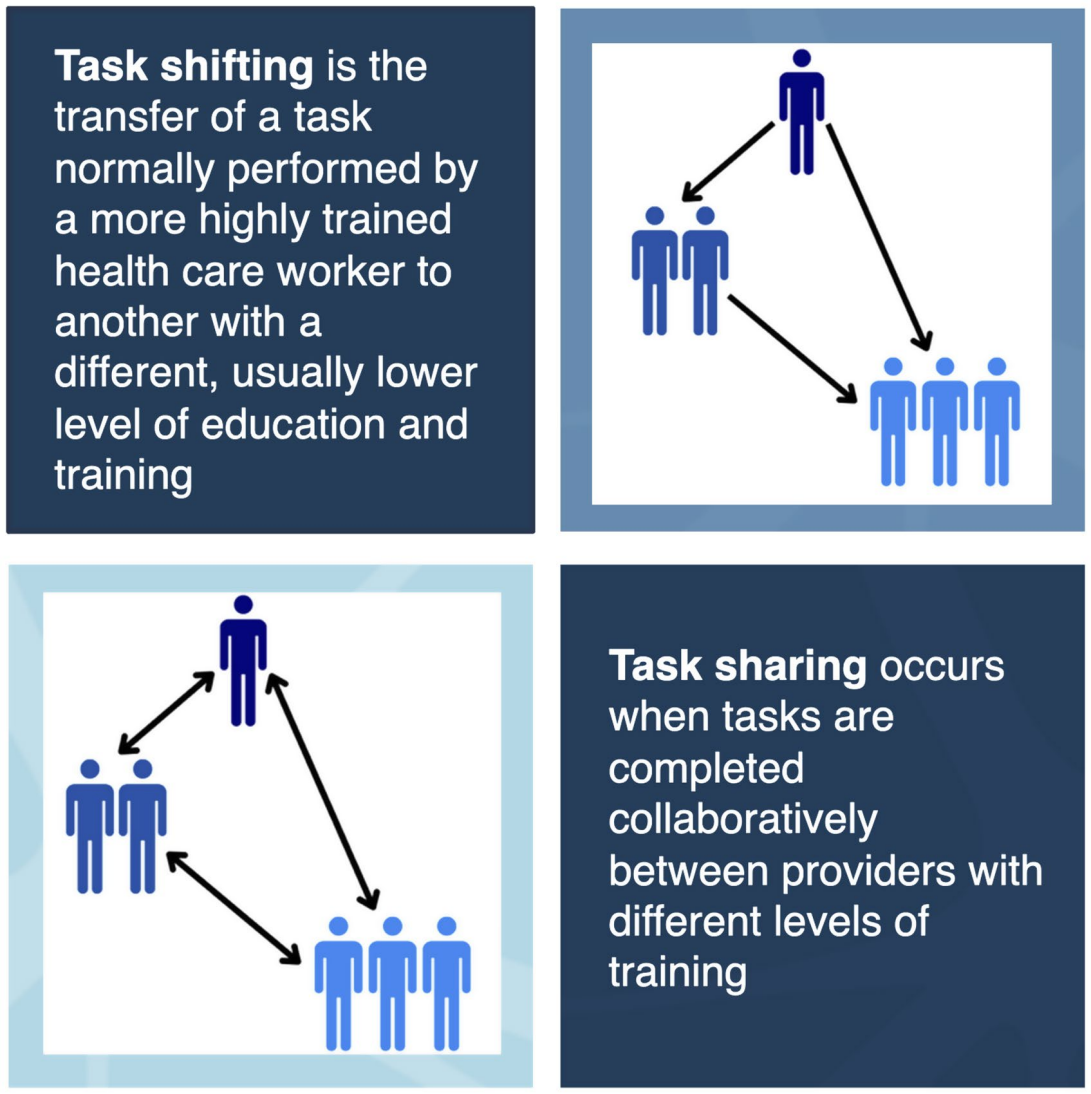
To overcome the shortage of specialists, task sharing and task shifting to non-clinician practitioners have been implemented with certain success in managing communicable and later non-communicable diseases. Task-shifting is the transfer of tasks typically performed by highly trained specialists to less specialised cadres after appropriate training. Task-sharing, by contrast, involves collaborative division of tasks among providers of different skill levels under supervision.

Both approaches are endorsed by WHO as efficient means to expand care coverage in low-resource settings (25). The success of these strategies is evidenced in the management of CDs: highly prevalent diseases that prompted the development of decentralised, NPCs and/or nurse-led, community-integrated care models (26). Task-shifting and task-sharing are not transitional compromises but may constitute long-term strategies for building more resilient healthcare systems, if appropriately regulated, supervised, remunerated, and integrated into functional referral systems. The investment in PC should be considered a complementary component of workforce development rather than a substitute for specialist services, with clearly defined scopes of practice and mechanisms to prevent excessive workload and ensure timely referral to higher levels of care. This approach could support the management of neurological diseases when embedded within formal policy frameworks. Without these safeguards, enthusiasm may fade and quality decline.

## From communicable to non-communicable diseases in primary care: a continuum of experience

The experience gained in the control of CDs across several countries of SSA provides a robust foundation for enhancing PC’s role as the gatekeeper for non-communicable diseases (NCDs), including neurological disorders. This was highlighted by the WHO in 2012 as a means of combating the concerning rise in the dual burden of HIV and NCDs. Over the past three decades, large-scale programmes addressing HIV, tuberculosis, and malaria have established effective models for screening, chronic care, adherence monitoring, and data management. Over the last two decades, these models have also developed an extensive infrastructure of training, supervision, and data collection, which is now supporting millions of patients. The same infrastructure is being increasingly repurposed for NCDs, including chronic diseases such as hypertension, diabetes, and, more recently, neurological conditions (27–29).

Building on these successes, the integration of HIV and NCD services has already shown measurable benefits. Combined clinics for HIV, hypertension, and diabetes in Uganda, Malawi, and Tanzania have improved follow-up rates, medication adherence, and stigma reduction, demonstrating that chronic care models can be safely and efficiently extended to multiple conditions (30). This approach optimises limited resources and promotes continuity, particularly when the same health workers provide integrated care within the community.

Clear-cut examples can be found in the informal settlement in Kibera in Kenya, where nurse-led management of hypertension and diabetes achieved sustained control in the majority of patients (31). A systematic review and meta-analysis of 63 studies on the effectiveness of task shifting in hypertension management in low- and middle-income countries showed a significant reduction in both systolic and diastolic blood pressure (32). In Malawi and the Central African Republic, NPCs have successfully undergone training in the diagnosis of epilepsy and treatment of stable cases (33). In Zambia, local task-sharing networks have helped improve efficiency, access and quality of care for neurological patients (34). Across Africa, stroke and rehabilitation programmes likewise highlight that decentralised neurological care can be effective, but only when training is linked to supervision, referral systems, and community follow-up. Reviews of stroke care in Africa identify recurrent barriers across the care pathway, including delayed presentation, limited rehabilitation capacity, high transport and treatment costs, and weak continuity between hospital and home (35). Community- and home-based models in South Africa have demonstrated that functional recovery is achievable in low-resource settings, yet they also reveal major system weaknesses, including poor discharge education, fragmented follow-up, low patient satisfaction, unmet assistive-device needs, and incomplete secondary prevention (36, 37). More broadly, rehabilitation studies from Ghana, Benin, Nigeria, and South Africa suggest that tele-rehabilitation, self-rehabilitation, task-specific physiotherapy, and vocational interventions can improve mobility, independence, and quality of life, but their sustainability depends on reliable supervision, referral guidance, rehabilitation staffing, and financial support for long-term care (38). Taken together, these experiences suggest that non-physician and community-based programmes can contribute meaningfully to stroke and neurological care, but only within organised systems that connect PC, preventative actions, rehabilitation, management of chronic conditions and specialist support over time (39, 40).

Overall, these experiences, from CD to NCDs and finally diving into the brain health framework, demonstrate that neurological task-shifting is feasible when supported by adequate training and supervision: they highlight how NPCs can serve as competent gatekeepers of brain health, capable of recognising common neurological presentations and initiating basic treatment or indicating referral. Specifically, neurology stands to benefit directly from this transformation since many neurological disorders share the same chronic trajectory as other NCDs. Embedding their management into PC structures allows earlier identification of at-risk individuals, timely treatment initiation, and sustained follow-up, reducing the overloading of tertiary hospitals. This continuum from CD to NCD management represents more than an administrative adjustment to improve care for headache and other chronic neurologic conditions—it embodies a paradigm shift in global neurology thinking. The determinants of infectious diseases and of neurological disorders often overlap with poverty, stigma, limited infrastructure, and restricted access to education. Addressing these shared determinants through integrated, life-course health systems bridges traditional divides and fosters sustainability.

Finally, this transformation also demands a cultural reorientation. The credibility once reserved for tertiary specialists must extend to trained PC professionals. When a well-trained NPC confidently manages a patient with neurological conditions, from headache or neuropathic pain to epilepsy, and communicates effectively, community trust in biomedical care increases and stigma decreases. Collaboration with local leaders can further reinforce this trust. Traditional and biomedical systems, working in synergy rather than competition, can enhance patient acceptance, continuity of care, and treatment adherence.

Through this continuum, PC evolves from being the endpoint of resource scarcity to becoming the epicentre of integrated, equitable neurology—rooted in the community and informed by decades of success in communicable-disease control.

## Education at the core of transformation

Education lies at the heart of the transformation of neurological care in Sub-Saharan Africa. The WHO IGAP explicitly identifies education as the primary mechanism for achieving equity in brain health. Within IGAP, education operates through four interconnected dimensions: awareness and stigma reduction, professional training, policymaker engagement, and inclusive health promotion. When developed together, these dimensions form the conceptual and operational backbone of neurology’s expansion into PC and community health.

However, achieving this vision remains constrained by the scarcity of training institutions across the region. The shortage of neurologists across the different SSA countries is largely driven by inadequate medical education infrastructure, including limited availability of medical schools and substantial faculty shortages due to poor remuneration and constrained career progression. Neurology training infrastructure remains unevenly distributed across SSA, although established specialist training programmes exist in several countries. These structural deficits intensify workforce gaps. Additionally, neurophobia—defined as difficulty in applying neuroscience in clinical contexts—is highly prevalent among SSA trainees and practitioners, as highlighted by several surveys among medical students who frequently express that “it’s too hard,” and cite challenges, including the volume and complexity of neurological knowledge, difficulties with neurological examination, and the breadth of differential diagnoses (41). It is primarily attributed to poor integration of basic and clinical neuroscience, perceived subject complexity, and inadequate teaching (42).

Only a few countries in SSA host structured neurology residency programmes, and even fewer offer formal long-term programmes to improve neurological education and training at the PC level. In addition, the shortage of neurologists perpetuates a vicious cycle: there are too few specialists to train the next generation, and too few academic institutions with the infrastructure to support such training. Simultaneously, NPCs, who provide most of the first-line care, receive limited or no exposure to neurology during their diploma or degree curricula. Their education typically focuses on internal medicine, obstetrics, or surgery, with minimal emphasis on brain health or chronic pain (43).

This educational deficit creates a twofold gap—quantitative and qualitative—that hinders the translation of global initiatives such as IGAP into tangible clinical capacity. Expanding the educational base thus requires not only creating more training programmes but also redefining how education is delivered. The evolution of medical teaching in Africa must progress from knowledge transmission to competence transformation. Traditional lecture-based learning, often reliant on memorisation, is insufficient for developing the diagnostic reasoning and critical thinking competencies required for neurological assessment.

Instead, competency-based and problem-oriented curricula should become the standard. (44) These models focus on observable skills, such as taking a detailed neurological history, performing a basic neurological examination, identifying “red flags,” and managing common conditions such as epilepsy and headache, rather than on time spent in the classroom. This challenge is captured by the well-known “know-do-gap”: the difficulty of translating acquired knowledge into routine clinical practice. Although this gap can occur across healthcare settings, its consequences may be particularly relevant in resource-constrained environments, where opportunities for supervision, continuing professional education, and clinical support are limited. (45–47) Integrating simulation-based training, case discussions, and mentorship-supported apprenticeship with repeated on-site visits to share work helps clinicians internalise concepts and adapt them to their local context. This approach is also key to develop a resilient tele-neurology system in low-resourced areas (48, 49).

Moreover, education must go beyond mere anatomy, algorithms and flowcharts. It has to strongly implement the ability to conduct an interview in outpatient and inpatient settings, as well as how to perform a physical evaluation, due to the peculiarity of the neurological and mental health framework and approach (44). It should also cultivate clinical reasoning, empathy, and communication skills: the hallmarks of quality neurological care. Training must blend classroom instruction with hands-on mentorship and community-based learning. Moreover, it helps to overcome the stigma surrounding certain conditions. Healthcare providers also tend to be afraid of evaluating patients with psychiatric or epileptic conditions due to their cultural heritage (50). Furthermore, many patients with chronic headache or pain have comorbid psychiatric conditions.

Tailoring education to local realities is crucial. In many SSA countries, power interruptions and limited connectivity challenge continuous online learning. Therefore, offline-compatible materials, such as printed manuals, portable digital libraries, and preloaded tablets, remain indispensable. At the same time, telemedicine and hybrid e-learning are becoming increasingly feasible. Emerging regional networks now support virtual consultations, continuing education, and remote supervision. Pilot tele-neurology initiatives in Kenya, Rwanda, and Malawi highlight that these tools can bridge distances between NPCs and supervising neurologists, therefore possibly improving diagnostic confidence and patient outcomes (48).

International partnerships play a catalytic role in this transformation. Collaborations between neurologic academic institutions, scientific societies, and national health systems, as well as regional PC programmes, allow the sharing of curricula, trainers, materials and standards, while ensuring local ownership. A fruitful example is the partnership between the International Headache Society^1^ and the DREAM program (see Box 1) in Malawi, for the promotion of headache medicine and neurology among NPC. Similarly, in the neurological field over the last decade, innovative partnerships were established between the Italian Society of Neurology (SIN) and DREAM in various SSA countries, involving also the C. Besta Neurologic Institute and the Global Health Telemedicine^2^ service. On a continental level, among the specialist-oriented initiatives, the long-established regional teaching course promoted by the European Academy of Neurology (EAN), in partnership with the African Academy of Neurology (AFAN), represents an important and strong program for the improvement of neurological education in SSA^3^. These partnerships also help develop a cadre of African trainers capable of leading regional educational initiatives, thereby ensuring sustainability.

Importantly, education should not flow in one top-down direction. The African experience in community-based, longitudinal, and multidisciplinary care offers valuable lessons for high-income countries now struggling with chronic disease management and health-system fragmentation. Global neurology must evolve from a unidirectional model of knowledge transfer to a reciprocal exchange of expertise, where experiences from resource-limited contexts enrich medical education worldwide.

Ultimately, education is not only the means to an end but the engine of equity. By strengthening neurological literacy across all levels of the healthcare system, education transforms territory and culture alike. In this respect, SIN has organised a structured teaching and training course for residents and young neurologists aimed at improving their knowledge on the mutated scenario of neurologic conditions and care models in SSA to help them properly operate in those scenarios^4^: global neurology requires tailored skills for globally more proactive neurologists (51).

BOX 1 **Focus on DREAM**

An Integrated Approach to Healthcare in Sub-Saharan Africa

Since its inception in 2002, the DREAM (Disease Relief through Excellent and Advanced Means https://www.dream-health.org/) program has been a cornerstone of public health intervention across SSA. Initially established by the Community of Sant’Egidio to address the escalating HIV/AIDS crisis, the program has evolved into a comprehensive, multi-faceted healthcare model. It currently operates across ten countries in SSA, shifting from a vertical approach to an integrated framework that combines HIV care with the management of other NCDs, thereby strengthening overall health systems.

The program’s impact is underscored by its scale and reach, with over 500,000 patients currently receiving regular, high-quality care from trained local healthcare professionals. A key driver of this success is the program’s commitment to technological innovation; since 2008, a dedicated telemedicine platform has bridged the gap between rural settings and specialized expertise, enabling local clinicians to consult with specialists remotely.

Central to the DREAM philosophy is the principle of sustainability through capacity building. Continuous, rigorous educational initiatives for both DREAM personnel and governmental healthcare providers form the core of the program. These efforts are specifically designed to expand clinical knowledge among physician and non-specialist care providers, empowering them to manage complex pathologies autonomously within their communities.

So far, 23 in-person teaching and training courses (52) on epilepsy and other highly prevalent neurologic diseases such as stroke and headache have been delivered to primary health care workers. Each course is followed by periods of shared work on the ground and training on the job in Malawi, Central African Republic, Mozambique, and the Democratic Republic of Congo.

## The IHS–DREAM Malawi initiative

### A case study for headache

The *International Headache Society–DREAM Malawi Initiative* illustrates how targeted education can recalibrate neurological care within a low-resource environment. The programme sought to strengthen local capacity in the diagnosis and management of primary and secondary headache (43, 53).

Two structured, in-person modular courses were delivered in 2022 and 2025, combining theoretical teaching with case-based learning and simulation exercises adapted to local clinical needs and aimed at building primary competence in neurology and headache care. The first course, held over 2 days in 2022, was delivered by IHS faculty and provided foundational training in headache medicine, with a strong emphasis on practical instruction, interactive discussion, and the application of knowledge to real-world clinical scenarios. The second course, conducted in 2025, was delivered by a faculty that also included five DREAM NPCs, who had been selected based on their successful completion of the first course and their prior training and clinical experience in other neurological fields, including epilepsy and stroke prevention. This model of progressive, peer-supported capacity building was already well established within DREAM (see Box 1) and had also been adopted in the IHS programme as a strategy to strengthen locally led education and support the long-term sustainability of the initiative within the healthcare system.

Overall, 51 learners took part in the two events^5^. They were mostly NPCs from primary and district hospitals, drawn from the national public service. As previously reported by Martinelli et al. 2025, in the evaluation of the educational programme, pre- and post-training assessments using a 33-item knowledge questionnaire demonstrated significant improvements in knowledge following the training, with a median increase of 11 points in 2022 and 18 points in 2025 (43). Crucially, several graduates later assumed teaching roles in their own districts, generating a train-the-trainers cascade that extended the programme’s educational reach through locally delivered activities, without requiring additional external funding for each subsequent teaching activity.

Although these results are encouraging, caution is warranted when extrapolating the Malawi experience to other settings across sub-Saharan Africa. Health systems within the region differ substantially in terms of workforce availability, governance, financing mechanisms, referral pathways, and access to diagnostic resources. Financial constraints represent an important determinant of neurological service capacity in SSA, affecting workforce availability, infrastructure, medicine procurement, referral systems, and opportunities for continuing professional education. However, national expenditure alone does not fully capture the capacity of health systems to deliver neurological care, as substantial variation exists in workforce distribution, financing mechanisms, and health-system organisation. Strengthening neurological care therefore requires not only increased investment, but also strategic allocation of available resources towards sustainable primary care, referral pathways, essential medicines, and locally led capacity development. Consequently, the effectiveness and scalability of educational interventions similar to the Malawi experience illustrated above are likely to depend on local contextual factors, including institutional commitment, integration within existing health services, and the availability of continued mentorship and supervision. Furthermore, while task-sharing models represent a pragmatic strategy to address the shortage of neurologists, they should not be regarded as a substitute for specialist training or broader health system strengthening. Sustaining improvements in neurology and headache care requires ongoing quality assurance, regular refresher training, monitoring of clinical outcomes, mechanisms to retain trained personnel, and incorporation of headache management into national health policies and professional curricula. The success of the Malawi initiative was facilitated by long-standing collaborations between local and international partners and by its integration into the established DREAM educational framework; replication in other provinces in Malawi or countries will therefore require careful adaptation to local priorities, resources, and health system structures rather than direct transfer of the model. These kinds of training programmes operate within finite resources and cannot achieve national coverage. Moreover, the Malawi IHS- DREAM programme was not designed or powered to demonstrate population-level scale-up, especially in a setting where striking workforce shortages remain. Still, the initiative provides a promising example of how contextually adapted educational interventions can contribute to capacity building and aligns closely with IGAP priorities on capacity building and equitable service distribution. The observed improvements in knowledge, together with participants’ subsequent involvement in teaching activities, suggest that such programmes may support the development of locally sustained educational capacity. However, whether these educational gains translate into improvements in clinical practice, patient care, and long-term service delivery remains to be established through prospective evaluation. The experience also highlights the potential importance of local engagement and ownership for sustaining educational activities within existing healthcare structures (54). The IHS-DREAM education program built its success also on the previous teaching and training courses on basic neurology and epilepsy delivered to DREAM’s NPCs thanks to pre-existing partnerships among DREAM, SIN and the IRCCS Besta Neurologic Institute (55).

## Discussion

### Towards a global, culturally rooted neurology

The treatment gap for headache disorders in SSA cannot be addressed through education alone, as it reflects broader health-system challenges. Increasing neurological knowledge is unlikely to produce sustainable improvements unless educational interventions are embedded within health systems that are geographically accessible, culturally acceptable, and supported by appropriate national health policies and referral pathways. This calls for a shift from viewing education as an isolated intervention to positioning it as an implementation tool within a broader, context-sensitive model of neurological care.

We propose a context-sensitive framework for neurological care in SSA based on three interconnected domains: territory, culture, and capacity. *Territory* refers to geographical accessibility and health-system organisation; *culture* to community engagement, trust, and local explanatory models of illness; and *capacity* to workforce development, education, and sustainable institutional support, particularly within PC. These pillars are interdependent: failure in any one of them limits the effectiveness of the others. Within this framework, education can serve as a bridge that links the three domains rather than an isolated intervention, but it must be accompanied by cultural legitimacy. Local leaders and other trusted community actors, including traditional healers, should therefore be engaged in dialogue, rather than excluded. Public trust can be strengthened through respectful dialogue that connects culturally rooted understandings of illness with appropriate healthcare pathways (56). Education is therefore both essential and insufficient. It develops the individual and institutional capacity needed to improve neurological care, but its impact depends on whether healthcare systems provide the resources, supervision, referral pathways, and policy support required to translate knowledge into practice. The objective should therefore be not simply to increase the number of trained providers, but to embed their skills within functional and sustainable care pathways.

A useful way to conceptualise this shift is to move from a *specialist-centred* model to a *system-centred* model of neurology. In specialist-centred frameworks, progress is measured by the number of neurologists trained or guidelines produced. In system-centred frameworks, progress is measured by whether patients can move through pathways of recognition, referral, diagnosis, treatment, and follow-up of neurological problems, starting from the very entry level: the *PC setting*. This distinction matters in SSA, where the principal failure is often not the absence of expertise somewhere in the system, but the absence of mechanisms that connect expertise to the communities that need it. Task-sharing therefore is effective only within a functioning tiered system in which more complex cases can access specialist assessment and higher-level services.

This distinction is especially relevant in headache care. Many current initiatives understandably focus on training healthcare professionals. However, while training healthcare professionals remains essential, education alone may be insufficient to achieve sustained improvements in access to care unless it is embedded within broader, context-sensitive health-system strategies. Without supervision, career pathways, essential medicines availability, referral networks, and institutional support, newly acquired competencies may not be retained or effectively applied. Likewise, increasing the number of trained providers alone cannot overcome geographical inequities or sociocultural barriers that influence healthcare-seeking behaviour. Future programmes should therefore be designed as integrated health system interventions, paying particular attention to the PC entry level, rather than stand-alone educational projects.

International neurological societies, guided by the IGAP framework, have both an opportunity and a responsibility to contribute to this transformation. Initiatives such as the *IHS–DREAM Malawi Programme* demonstrate that educational interventions, when aligned with local realities and supported by existing CD infrastructures, can create tangible change (54). In order to promote this change toward global neurological/headache care, these educational efforts need to be matched by appropriate institutional policies. The global IHS practice recommendations for acute and preventive migraine treatment are a fruitful example, deliberately structured around essential and optimal levels of care so that they remain applicable even in resource-constrained settings (57, 58). However, guidelines alone cannot close the treatment gap. International survey data show major inequities in access to headache medicines, especially in Africa, and even drugs already included in the WHO essential medicines list are not consistently available (59).

From a policy perspective therefore, five reforms are likely to have the greatest impact: (i) embedding headache care within national neurological and primary healthcare strategies; (ii) establishing sustainable train-the-trainer programmes linked to local medical and academic institutions; (iii) ensuring reliable access to essential headache medicines through national procurement systems; (iv) developing locally led implementation research capable of evaluating context-specific models of care rather than replicating approaches developed in high-income settings and (v) expanding telemedicine-enabled supervision and referral support. Collectively, these reforms would move the focus from episodic educational projects towards resilient neurological systems capable of adapting to the diversity of healthcare environments across SSA.

For this reason, headache advocacy in SSA must extend beyond education to action to improve access to effective acute and preventive therapies (60). This agenda is now advancing further through work to expand the WHO essential medicines list for headache disorders. Because more than 150 countries use the WHO list to inform their own national essential medicines lists, additions at that level can have direct policy relevance for low- and middle-income settings (61). The IHS has contributed to this process, working with partner organisations to submit new proposals supported by evidence reviews and cost-effectiveness (61).

This also has implications for global neurology as a discipline. The field should move beyond short-term outreach and toward reciprocal capacity-building. High-income training programmes should prepare neurologists for work in low-resource settings through education in cultural humility, syndromic diagnosis, and resource-stratified practice (62). At the same time, African clinicians should lead the design of regional research, education, and implementation strategies. The most durable advances will come from models in which expertise is not imported episodically but developed locally and sustained institutionally.

Taken together, these considerations suggest that the next phase of neurological development in SSA should not be defined primarily by expanding education, but by repositioning education within a broader system of implementation, starting from PC. Neurological care will become more equitable only when workforce development, task sharing, medicine access, cultural engagement, and national policy are pursued as components of a single implementation strategy.

## Conclusion

The neurological challenges in SSA are not primarily technical but systemic, cultural, and contextual.

We therefore advocate a shift from disease-centred interventions to context-centred implementation, in which education, health policy, workforce development, access to essential medicines, and community engagement evolve together. Consolidating primary care is central to this transition because most patients first encounter the health system at that level.

The IHS–DREAM experience in Malawi illustrates this principle: strengthening headache medicine through locally adapted education can serve as an entry point for broader neurological capacity building. More specifically, it demonstrates how partnerships between international scientific societies and well-established local healthcare programmes can create the conditions for effective task-sharing by empowering primary healthcare providers with context-appropriate knowledge, skills, and referral pathways.

Education is therefore both essential and insufficient. It is the mechanism through which individual and institutional capacity is developed, being the common denominator linking the IGAP vision, task-sharing strategies, and cultural transformation. However, its impact depends on whether it is integrated into systems capable of translating knowledge into timely access, continuity of care, and effective treatment. By embedding neurology and headache medicine within the fabric of primary care and aligning them with the successful paradigms of chronic disease management, SSA can establish a sustainable framework for brain health. In doing so, it may also offer the global neurological community a model of how equity, adaptability, and partnerships between scientific societies and local healthcare providers can redefine the delivery of neurological care.

The future of global neurology and headache medicine requires moving beyond the episodic transfer of specialist expertise towards sustained investment in resilient, context-sensitive systems that integrate locally led capacity building, equitable access to essential medicines, and culturally grounded implementation.

## Data Availability

Publicly available datasets were analyzed in this study. This data can be found at: https://doi.org/10.1177/25158163251396915.

## Footnotes

1
https://ihs-headache.org/

2
https://ghtelemedicine.org/

3
https://www.ean.org/learn/educational-events/sub-saharan-africa

4
https://www.neuro.it

5
https://ihs-headache.org/wp-content/uploads/2025/03/ROPE-IHS-newsletter-report-2025.pdf
